# A novel reflex cough testing device

**DOI:** 10.1186/s12890-017-0365-y

**Published:** 2017-01-18

**Authors:** Kazunori Fujiwara, Katsuyuki Kawamoto, Yoko Shimizu, Takahiro Fukuhara, Satoshi Koyama, Hideyuki Kataoka, Hiroya Kitano, Hiromi Takeuchi

**Affiliations:** 1 Department of Otolaryngology, Head and Neck Surgery, Faculty of Medicine, Tottori University, 36-1, Nishimachi, Yonago, 683-8504 Japan; 2 Department of Rehabilitation, Tottori University Hospital, 36-1, Nishimachi, Yonago, 683-8504 Japan

**Keywords:** Aspiration pneumonia, Silent aspiration, Reflex cough test, Peak cough flow, Involuntary cough

## Abstract

**Background:**

The reflex cough test is useful for detecting silent aspiration, a risk factor for aspiration pneumonia. However, assessing the risk of aspiration pneumonia requires measuring not only the cough reflex but also cough strength. Currently, no reflex cough testing device is available that can directly measure reflex cough strength. We therefore developed a new testing device that can easily and simultaneously measure cough strength and the time until the cough reflex, and verified whether screening with this new instrument is feasible for evaluating the risk of aspiration pneumonia.

**Methods:**

This device consists of a special pipe with a double lumen, a nebulizer, and an electronic spirometer. We used a solution of prescription-grade L-tartaric acid to initiate the cough reflex. The solution was inhaled through a mouthpiece as a microaerosol produced by an ultrasonic nebulizer. The peak cough flow (PCF) of the induced cough was measured with the spirometer.

The 70 patients who participated in this study comprised 49 patients without a history of pneumonia (group A), 21 patients with a history of pneumonia (group B), and 10 healthy volunteers (control group).

**Results:**

With the novel device, PCF and time until cough reflex could be measured without adverse effects. The PCF values were 118.3 ± 64.0 L/min, 47.7 ± 38.5 L/min, and 254.9 ± 83.8 L/min in group A, group B, and the control group, respectively. The PCF of group B was significantly lower than that of group A and the control group (*p* < 0.0001), while that of group B was significantly lower than that of the control group (*p* < 0.0001). The time until the cough reflex was 4.2 ± 5.9 s, 7.0 ± 7.0 s, and 1 s in group A, group B, and the control group, respectively. This duration was significantly longer for groups A and B than for the control group (A: *p* < 0.001, B: *p* < 0.001), but there was no significant difference between groups A and B (*p* = 0.0907).

**Conclusion:**

Our newly developed device can easily and simultaneously measure the time until the cough reflex and the strength of involuntary coughs for assessment of patients at risk of aspiration pneumonia.

**Electronic supplementary material:**

The online version of this article (doi:10.1186/s12890-017-0365-y) contains supplementary material, which is available to authorized users.

## Background

Pneumonia is a common event after strokes and is associated with morbidity and mortality in stroke patients. It reportedly occurs in one-third of all stroke victims and is the most common respiratory complication in this population [1]. The risk factors for aspiration pneumonia include silent aspiration caused by old age and neuromuscular disorders, but not cerebral stroke. The identification of patients who are at risk of developing pneumonia is thus important in terms of morbidity and mortality as well as cost of care.

The videofluorographic swallowing study (VFSS) is the definitive test to identify aspiration and other swallowing abnormalities [2]. Previous studies have shown that aspiration observed on VFSS indicates a risk of developing aspiration pneumonia. The laryngeal cough reflex protects the laryngeal aditus from significant aspiration of food, fluids, and secretions, and reduces the risk of aspiration pneumonia [3, 4]. However, VFSS is not routinely used to assess the laryngeal cough reflex. Dysphagia and the cough reflex are separate physiologic and neurologic issues and should therefore be independently assessed. Furthermore, several screening tests, including the water test, food test, and saliva swallowing test, cannot detect silent aspiration in patients without a cough reflex.

Previous research has suggested that adding a cough sensitivity test to clinical swallowing evaluations has the potential to reduce the risk of pneumonia after stroke. A clinical guideline for cough assessment produced in 2007 by the European Respiratory Society Task Force highlighted the lack of standardization of cough testing protocols using cough-stimulating agents [5]. In the reflex cough test (RCT) the patient inhales citric acid and tartaric acid from low to high concentrations and the presence of a cough is assessed. Studies have shown that the inhalation of tartaric acid stimulates coughs in normal subjects [3, 6, 7], and that the RCT is useful for the detection of silent aspiration [8, 9]. In these studies, nebulized tartaric acid was shown to be safe and did not produce systemic or adverse side effects. However, the RCT results were evaluated in terms of the presence and number of coughs, but not cough strength. Voluntary cough strength can be measured with a spirometer, but up to this point no devices have been able to measure involuntary cough strength as part of the RCT. Another drawback of the RCT is the subjective evaluation of cough strength by the examiner, because it is important to determine whether the cough generated by the stimulating agent is effective, and whether the subject can cough up secretions.

To overcome these shortcomings, we developed a device to quantify the cough reflex. This paper describes the use of an objective examination system, called the modified RCT (mRCT), to quantify both a tartaric-acid-stimulated involuntary cough and the time until the cough reflex. The study also investigated whether screening with this new instrument would be feasible for evaluating the risk of aspiration pneumonia.

## Methods

### Apparatus for the mRCT

**Fig. 1:**
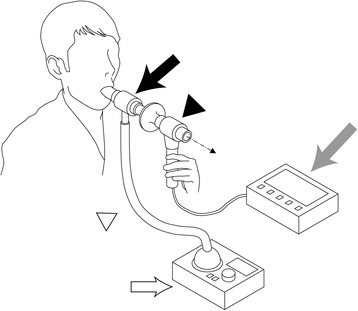
Schematic diagram of modified reflex cough test using our new equipment. The new cough reflex testing device consists of a mouthpiece, tube, filter, spirometer (*gray arrow*), and ultrasound nebulizer (*white arrow*). The tube is designed as a double pipe (*black arrow*) with ten holes between the inner and outer pipes. A corrugated tube (*white triangle*) from the ultrasound nebulizer is connected to the outer pipe, and the spirometer sensor is attached to the inner pipe (*black triangle*). The microaerosol emitted from the ultrasound nebulizer fills the outer pipe, and is administered to the oral and airway epithelia through the holes and mouthpiece. The cough then passes through the inner pipe to the spirometer sensor

The new mRCT device consists of a mouthpiece, tube, filter, spirometer IH-105 (Chest M.I., Inc., Tokyo, Japan), and ultrasound nebulizer UN-703 (Alfresa Pharma Corporation, Osaka, Japan). The tube was designed as a double pipe with 10 holes between the inner and outer pipes, which are made of rigid plastic material. We designed this instrument and ordered the resin from a resin-processing company. A corrugated tube is connected from the ultrasound nebulizer to the outer pipe, and the spirometer sensor is attached to the inner pipe. The microaerosol emitted by the ultrasound nebulizer fills the outer pipe, and is administered to the oral and airway epithelia through the holes and mouthpiece. The cough then passes through the inner pipe to the spirometer sensor, which shows the peak cough flow (PCF) value on the monitor (Fig. 1). The device can thus easily quantify the flow of an involuntary cough.

### mRCT

The RCT stimulates cough receptors in the vestibule of the larynx and initiates the laryngeal cough reflex [3, 6, 10–12]. The mRCT in this study used a 20% solution of prescription-grade l-tartaric acid dissolved in 2 mL of sterile normal saline [8]. The solution was placed in an ultrasound nebulizer and inhaled as a microaerosol. During the inhalation, the subject’s nose was pinched closed. The mRCT was administered by either a speech therapist or an otorhinolaryngologist at the bedside or at the ENT clinic of Tottori University Hospital. Subjects were instructed to exhale, to position the mouthpiece appropriately, and then to inhale sharply and deeply and to hold their breath after inhaling. To prevent leakage around the mouthpiece, subjects were asked use their lips to pull it toward the back of the throat. The examiner held the device to prevent it from falling. The mRCT was considered to be complete when either a cough response was elicited or the subject failed to respond within 30 s after the start of the inhalation. The spirometer showed the reflex cough strength as the PCF on the display. The time from the start of tartaric acid administration until the cough reflex was defined as the time until cough reflex. Both this time and the PCF were recorded. If there was no cough reflex, the recorded PCF value was 0 ml/min. For each subject this test was performed once per day to prevent test-induced changes to the sensitivity threshold of the airway mucosa from influencing a subsequent test. If the examination was suboptimal, it was repeated on another day. Furthermore, patients who had experienced aspiration pneumonia were examined after they had completely recovered.

To prevent infection, the double pipe was soaked in glutaral and rinsed under running water. Furthermore, the filter, corrugated tube, and mouthpiece were all disposable.

### Statistical analysis

All values are presented as mean ± SD. For statistical analyses, the Mann-Whitney *U* test was used to evaluate to evaluated the differences of the PCF between different groups of patients, and the log-rank test was used to assess the time until the cough reflex. The Mann-Whitney *U* test was used to statistically analyze the differences in age and BMI between each group.

## Result

### Patients

Between April 2013 and March 2014, 70 patients participated in our clinical trial of the mRCT. All had dysphagia as a subjective symptom and were referred for an examination of swallowing function due to neuromuscular disease, cerebral stroke, or aspiration pneumonia. The Tottori University institutional review board approved the use of the mRCT with tartaric acid for the screening of silent aspiration. Informed consent was obtained from all patients. VFSS, video endoscopy, and manometery, but not the mRCT, were performed for the evaluation of swallowing function. Patients with trismus or disordered consciousness were excluded from the trial. Before the mRCT, we determined patients’ past history of pneumonia by asking them directly and checking their medical records. The 49 patients without previous pneumonia were classified as group A, and 21 with such a history comprised group B. Group A consisted of 36 males and 13 females with an average age of 69.7 ± 11.0 years and an average body mass Index (BMI) of 20.7 ± 3.8. Group B consisted of 11 males and 10 females with an average age of 71.7 ± 17.0 years and an average BMI of 18.2 ± 3.3. Additionally, 10 healthy volunteers participated in the clinical study as the control group; these individuals had an average age of 36.6 ± 19.9 and an average BMI of 20.8 ± 1.4. Although we found no significant difference between groups A and B in terms of age, they differed significantly in terms of BMI. The healthy controls were significantly younger than the two disease groups. The primary diseases of all patients are shown in Table 1. Two years after the study concluded, 4 patients (8.1%) in group A developed aspiration pneumonia. In contrast, 14 patients (66.7%) in group B developed aspiration pneumonia, and 4 (19.0%) underwent laryngeal separation surgery. VFSS identified dysphasia in 29 patients in group A (13 aspiration, 10 penetration, 6 pooling in the pharynx) and in 17 patients in group B (14 aspiration, 2 penetration, 1 pooling in the pharynx). All patients completed the mRCT without any adverse effects or complications resulting from its administration. The mRCT was performed as previously described and the PCF and the time until the cough reflex were recorded.

**Table 1:** Primary disease of all patients

Group A		Group B	
Parkinson disease	8	Parkinson disease	5
hypopharyngeal cancer	6	cerebral infarction	3
cerebral infarction	5	multiple system atrophy	3
multiple system atrophy	4	amyotrophic lateral sclerosis	2
amyotrophic lateral sclerosis	2	chordoma	1
laryngeal cancer	2	dementia	1
oropharyngeal cancer	2	encephalomyelitis	1
progressive supranuclear palsy	2	COPD	1
Wallenberg syndrome	2	Gaucher disease	1
Alzheimer	1	myasthenia gravis	1
aortic aneurysm	1	pneumonia	1
asthma	1	recurrent laryngeal nerve palsy	1
dementia	1		
dermatomyositis	1		
maxillary cancer	1		
mitochondrial myopathy	1		
motor neuron disease	1		
muscular sarcoidosis	1		
myasthenia gravis	1		
myotonic dystrophy	1		
pontine infarction	1		
recurrent laryngeal nerve palsy	1		
schizophrenia	1		
spinocerebellar degeneration	1		
thalamic hemorrhage	1		

### mRCT

This system could measure the PCF and time until cough reflex without any adverse effects. The test took less than 30 s and the preparation time was less than 2 min. The test was performed by one examiner without assistance. Thirteen patients were evaluated in the supine position and 57 patients and the 10 healthy volunteers were evaluated in the sitting position. All three groups of patients were included in the analysis.

### PCF

**Fig. 2:**
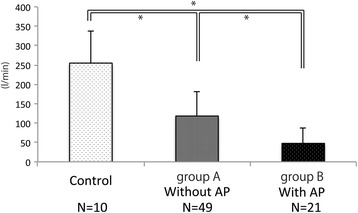
Peak cough flow of controls and patients with and without AP. The PCF of group B (*with AP*) was significantly lower than that of group A (*without AP*) and the control group, while that of group A was significantly lower than that of the control group. (* *p* < 0.0001)

During the mRCT, one patient exhibited no cough reflex within 30 s after the start of inhalation. The PCF of group A was 118.3 ± 64.0 L/min, that of group B was 47.7 ± 38.5 L/min, and that of the control group was 254.9 ± 83.8 L/min. The PCF of group B was significantly lower than that of group A and the control group (*p* < 0.0001), while that of group B was significantly lower than that of the control group (*p* < 0.0001) (Fig. 2). The PCF values of patients with and without development of aspiration pneumonia after the study concluded were 0.93 ± 0.76 and 1.87 ± 1.11, respectively, which was significantly different (*P* < 0.0002). In terms of the correlation between VFSS and PCF results, the PCF of patients with normal VFSS finding was 1.7 +/− 1.2 L/min, and that of patients with dysphagia was 1.5 +/− 1.0 L/min. There was no significant difference between patients with and without dysphagia.

### Time until cough reflex

**Fig. 3:**
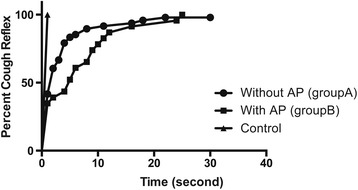
Time until cough reflex in controls and patients with and without AP. The time until cough reflex was significantly longer in groups A (*without AP*) and B (*with AP*) than in the control group (*A*: *p* < 0.001, *B*: *p* < 0.001), but there was no significant difference between groups A and B (*p* = 0.0907)

The time until the cough reflex was 4.2 ± 5.9 s in group A, 7.0 ± 7.0 s in group B, and 1 s in the control group. The time until the cough reflex was significantly longer in groups A and B than in the control group (A: *p* < 0.001; B: *p* < 0.001), but there was no significant difference between groups A and B (*p* = 0.0907) (Fig. 3).

## Discussion

Silent aspiration is defined as aspiration without coughing or other obvious distress, and has been linked to an increased prevalence of pneumonia and mortality [13, 14]. The risk of pneumonia was found to be increased 10-fold in patients with profound aspiration and 13-fold in those with silent aspiration [15]. However, patients with silent aspiration have no symptoms, including coughing, and dysphagia is therefore more likely to be identified late in disease development. Early identification of silent aspiration and prevention of pneumonia are thus essential in view of the high mortality associated with these conditions.

VFSS is the definitive test for identification of aspiration and other swallowing abnormalities, but it cannot be performed at every institution. This makes swallowing screening tests essential. These tests consist of the water screening test, food test, saliva swallowing test, and RCT. Tohara et al. reported that the water swallowing test (3 ml of water) and the food test (4 g of pudding) had a sensitivity of 90% and specificity of 56% [16], while Nishiwaki et al. found that the sensitivity of the saliva-swallowing test was 28% and the specificity was 76% [17]. However, since patients with silent aspiration have difficulty coughing, a simple screening test cannot be used to detect their condition in the clinical setting. The RCT is reportedly the method of choice for detecting silent aspiration, and uses citric acid and tartaric acid to trigger the cough [9].

The RCT using citric acid demonstrated a diminished cough reflex in patients with aspiration pneumonia [9]. An impaired cough reflex may play a role in the pathogenesis of aspiration pneumonia, and it was suggested that the early detection of a diminished cough reflex in patients with a predisposition to aspiration helped prevent aspiration pneumonia [9]. A previous study reported that the RCT fail rate for patient cohorts with suspected dysphagia was 25% for VFSS and 23% for fiber optic endoscopic evaluation of swallowing [18].

However, the RCT using citric acid takes a long time, and the procedure and decision criteria are complicated, thus making it difficult to use this test to examine patients in debilitated condition. The RCT using tartaric acid is an alternative method that is simple and quick and can thus be more appropriate for such patients. Addington et al. reported that using the tartaric acid RCT after a neurological event was essential for determining the appropriate clinical treatment plan for prescription of food, fluids, and medications. Moreover, the RCT helps to stratify pneumonia risk and improves outcomes by reducing morbidity, mortality, and cost [8]. One major problem with the conventional RCT method is that cough strength is subjectively evaluated by the examiner, thus results may differ among providers.

We developed an objective measuring device to simultaneously quantify cough strength and the time until the cough reflex occurs. This equipment is a novel device that can assess the relationship between aspiration pneumonia and PCF, the latter measured with the RCT using tartaric acid. The merits of our device are that it can quantify the triggered involuntary cough and measure the time until the cough reflex; there are no adverse reactions, and the examination takes only a short time.

An electromyography-based RCT system that can also evaluate the risk of aspiration pneumonia was patented in the United States in 2004 (https://www.google.com/patients/us20040181161). However, the use of electromyography to measure cough strength is an indirect approach. If patients have upper respiratory tract conditions that impair effective cough expulsion, such as laryngeal paralysis and head and neck cancer, electromyography cannot accurately measure cough strength. In contrast, our device is able to directly evaluate cough strength.

The results of our screening test showed that there was a significant difference in the PCF of patients with and without a history of pneumonia. Thus, the PCF value may indicate the risk of pneumonia and suggests that our equipment permits objective quantification and evaluation.

Hammand et al. reported that peak flow during the inspiration phase and the sound pressure level of voluntary cough was significantly impaired in severe aspirators as compared with non-aspirators [19]. Beck et al. evaluated voluntary cough and found that in patients with severe respiratory muscle insufficiency and a PCF of less than 160 L/min, decannulation was unsuccessful due to difficulty in keeping the airway clear after extubation and decannulation [20]. Lasserson et al. reported that PCF of involuntary cough was less than that of voluntary cough, explaining that this was related to differences between the two types of cough in terms of the functional organization of muscle activation [21]. Tartaric acid is thought to induce coughing by stimulating irritant receptors and C-fiber receptors [5]. Unlike a voluntary cough, the stimulus that induces an involuntary cough is delivered to the cough center, located in the nucleus tractus solitarius of the medulla oblongata, via sensory nerves ending through the vagus nerve afferent pathway sensory nerve ending, and not under cerebral cortex control. As explained above, quantification and evaluation of involuntary cough are indispensable for the assessment of aspiration. Since the novel measurement system developed in this study overcomes various difficulties and can measure the PCF of involuntary cough, it can provide a realistic assessment of the risk of aspiration pneumonia.

Our device can also measure the sensitivity threshold of the airway mucosa. Moreover, its use showed that there was a significant difference in the time until cough reflex between normal subjects and patients with dysphagia, making it possible to determine the relationship between the time until cough reflex and dysphagia.

There was no significant difference in the time until cough reflex between patients with and without a previous history of pneumonia. There are actually two distinct types of reflexive, non-voluntary cough with different underlying neurological mechanisms: a primary cough reflex occurring at the level of the vocal folds, often referred to as the laryngeal cough reflex, and a deeper tracheobronchial cough reflex that tends to be delayed and less productive [22]. The receptors in the larynx and trachea are extremely sensitive to mechanical stimuli, with very rapid adaptation. Deeper into the airways, the receptors become more chemosensitive and less mechanosensitive. The aerosol can thus penetrate deeper into the lung and stimulate the more chemosensitive cough receptors there. In other words, the chemosensitive stimulation of the tartaric acid aerosol differs from the mechanosensitive stimulation of food and saliva. Thus, the time until the cough reflex may not be accurately assessed by mechanosensitive stimulation.

VFSS is the gold standard for evaluating swallowing. However, VFSS showed no significant differences in peak flow between patients with and without dysphagia. Since VFSS evaluates swallowing dynamics, it is suggested that there was no relationship between swallowing dynamics and cough strength, and the cough reflex test should be used in combination with VFSS to compensate for limitations of each test.

There are certain limitations to our study. First, patients with trismus or disordered consciousness were excluded from this study because the device was not suitable in these populations. Further development of our device is required to generalize its use. Second, PCF and time until cough reflex were retrospectively evaluated based on past history of aspiration pneumonia, and no prospective cases of aspiration pneumonia were studied in this report. In the near future, we plan to prospectively evaluate the risk of aspiration pneumonia using our novel device. Third, since differences in cough reflex may be age related rather than disease specific, further work will need to be undertaken in the future with age-matched controls to ensure this is not the case. Future studies of the diagnostic accuracy of this device will ideally recruit participants using a single-gate design in order to more accurately identify the test characteristics that are applicable to clinical practice. It has been demonstrated that increasing age contributes far more to reduced local sensitivity than do neuromuscular disorders, which explains the higher prevalence of dysphagia in older patients [23]. Moreover, several studies have shown that silent aspiration is associated with advancing age [24–26]. This means that there are many patients at risk of silent aspiration, and if a method could easily assess this risk it would be in great demand. Furthermore, if our method becomes more prevalent, it will make it easier for patients at risk of silent aspiration to gain access to specialized medical care. We would like to miniaturize and simplify our device to make it available not only to medical staff but to any qualified personnel.

## Conclusion

Our newly developed device can easily and simultaneously measure the time until the cough reflex and the power of involuntary cough, without any adverse effects, for assessment of patients at risk of aspiration pneumonia. This constitutes the first system that can directly measure the strength of involuntary cough.

## Additional file

Additional file 1: Modified reflex cough test and videofluoroscopy test. The 49 patients without previous pneumonia were classified as group A, and 21 with such a history comprised group B. Additionally, 10 healthy volunteers participated in the clinical study as the control group. The peak cough flow (PCF) of the induced cough and time until cough reflex was measured with the spirometer. Dysphasia was evaluated for the patients by VFSS. (XLSX 54 kb)

## Abbreviations

PCF: Peak cough flow
RCT: Reflex cough test
VFSS: Videofluorographic swallowing study
